# Paper-Based Biosensor for the Detection of Sepsis Using MMP-9 Biomarker in FIP Mice Model

**DOI:** 10.3390/bios13080804

**Published:** 2023-08-11

**Authors:** Nuha Khalid Alekhmimi, Zeyad Raddadi, Abdulelah A. Alabdulwahed, Shimaa Eissa, Dana Cialla-May, Jürgen Popp, Khaled Al-Kattan, Mohammed Zourob

**Affiliations:** 1 Department of Chemistry, Alfaisal University, Al Zahrawi Street, Al Maather, AlTakhassusi Rd, Riyadh 11533, Saudi Arabia; nalekhmimi@outlook.com; 2 Institute of Physical Chemistry and Abbe Center of Photonics, Friedrich Schiller University, 07745 Jena, Germany; dana.cialla-may@leibniz-ipht.de (D.C.-M.); juergen.popp@ipht-jena.de (J.P.); 3 Cell Therapy and Immunobiology Department, King Faisal Specialist Hospital and Research Center, Riyadh 13541, Saudi Arabia; zraddadi@kfshrc.edu.sa; 4 Saudi Food and Drug Authority, Riyadh 13513, Saudi Arabia; aaabdulwahed@sfda.gov.sa; 5 Department of Chemistry, Khalifa University of Science and Technology, Abu Dhabi P.O. Box 127788, United Arab Emirates; shimaa.eissa@ku.ac.ae; 6 Advanced Materials Chemistry Center (AMCC), Khalifa University of Science and Technology, Abu Dhabi P.O. Box 127788, United Arab Emirates; 7 Leibniz Institute of Photonic Technology, Member of Leibniz Health Technologies, Member of the Leibniz Centre for Photonics in Infection Research (LPI), Albert Einstein Straße 9, 07745 Jena, Germany; 8 College of Medicine, Alfaisal University, Al Zahrawi Street, Al Maather, Al Takhassusi Rd, Riyadh 11533, Saudi Arabia; kkattan@alfaisal.edu

**Keywords:** sepsis, biomarkers, matrix metalloproteinase, MMP-9, early detection, sepsis diagnosis, paper-based biosensor

## Abstract

Sepsis is an immune response to a microbial invasion that causes organ injury and dysfunction due to a systemic inflammatory response. Sepsis is a serious, life-threatening condition and a widely recognized global health challenge. Given its high death rate, it is critical to diagnose sepsis and start treatment as early as possible. There is an urgent need for a sensitive and rapid screening method for detecting sepsis. In this study, we investigated the use of MMP-9 as a biomarker for sepsis. A colorimetric paper-based biosensor was used for the detection of MMP-9 utilizing peptide-magnetic nanoparticle conjugates. The method is based on the cleavage of the MMP-9-specific peptide by the protease leading to the detaching of the magnetic beads from the sensor surface and changing of color. A fecal intraperitoneal (FIP) challenge was used to induce sepsis in mice, and an MMP-9 secretion was measured by taking blood and Bronchoalveolar Lavage (BAL) fluid samples at 1 h, 2 h, 4 h, and 20 h (early sepsis) post-challenge intervals. The results of the paper-based sensor for the detection of MMP-9 levels in blood samples and BAL samples were compared with ELISA and Western Blot. We found that both blood and BAL levels of MMP-9 increased immediately and could be detected as early as 1 h in FIP mice post-challenge. Our work adds evidence to the assertion that MMP-9 is a reliable biomarker for the detection of sepsis at early stages.

## 1. Introduction

Sepsis is a life-threatening clinical manifestation of infection and represents a major cause of hospitalization worldwide, with a global mortality rate of 42% in 2020 [1]. Sepsis is the leading cause of death in most intensive care units (ICUs) and is a major problem in the intensive care units of Saudi Arabian hospitals. In fact, between 2002 and 2017, 46.8% of all patients admitted to a tertiary ICU in Saudi Arabia had sepsis, and 31% had septic shock [2]. Annual deaths from sepsis are estimated to be approximately 5.3 million globally [3]. If not managed promptly, sepsis develops into septic shock, multiple organ failure, and death. Despite advances in sepsis management, therapeutic procedures, and antibiotic treatments, sepsis remains a major healthcare concern worldwide.

At a biological level, sepsis is a systemic inflammatory response characterized by a cytokine-mediated hyper-inflammatory phase that is immediately followed by an immune-suppressive phase, which induces immune response deregulation including apoptotic depletion of immune cells [4,5], increased T-cell activity, a massive loss of lymphocytes, and cellular exhaustion. These effects ultimately lead to infections that the patients cannot overcome, causing septic shock and death [6]. One of the most common presentations of a patient with advanced sepsis is acute lung injury (ALI) and acute respiratory distress syndrome [7]. The early recognition of sepsis with timely interventions and early administration of antibiotics is critical for increasing the likelihood of survival for sepsis patients.

One of the major approaches to improving sepsis identification and diagnosis is the development and use of reliable predictive biomarkers for sepsis. Being able to accurately identify biomarkers associated with the onset of the septic disease process will help clinicians identify patients at higher risk of sepsis, thereby allowing closer monitoring and early treatment to reduce mortality. Clinical scores typically used in emergency departments include systemic inflammatory response syndrome and the Sequential Organ Failure Assessment [8]. One of the major challenges in developing a biomarker for sepsis in humans is the wide variability in its presentation between subjects. Patients are admitted to the ICU at different stages of the septic disease process, and they typically display varying signs and severity of symptoms depending on the individual case and circumstances of the initial infection. However, animal models offer a robust and reliable model for scientists to investigate disease pathogenesis, and they offer a framework for identifying the potential biomarkers of a particular disease. Understanding the kinetics of disease development is crucial to identify clinically relevant biomarkers, especially for a complex disease such as sepsis, which has different levels of presentation and can rapidly lead to death.

There is strong evidence to suggest that matrix metalloproteinases (MMPs) and their tissue inhibitors are promising predictive biomarkers for the diagnosis and prognosis of disease [9,10]. MMPs are a family of zinc-dependent endopeptidases that are involved in processes that remodel the extracellular matrix (ECM), including leukocyte migration and the repair of damaged tissue [9,11]. MMP-9 plays a central role in the body’s immune system. During the course of many diseases, there is a rapid increase in the MMP-9 expression by the body’s [12] leukocytes, neutrophils, monocytes, macrophages, and lymphocytes [13,14,15,16]. The cell walls of Gram-negative bacteria lipopolysaccharide (LPS) have been shown to induce the release of MMP-9 via neutrophils and monocytes in sepsis patients. Recently, it has been suggested that MMP-9 blocking could be exploited as a possible therapeutic approach for sepsis [10,17,18]. 

MMP-9 is elevated in patients with acute lung injury (ALI) and acute respiratory distress syndrome (ARDS), which correlates closely with lung injury severity [19,20,21] and supports the assertion that MMP-9 plays a major role in the pathophysiology of sepsis [10,17,22]. A single-nucleotide polymorphism (SNP) at position-1562 (C/T) in the MMP-9 gene has been associated with differential MMP-9 expression and increased mortality in sepsis patients [23]. Studies have demonstrated that MMP-9 levels are significantly elevated in patients with acute sepsis and are associated with short-term mortality risk [24,25,26]. Thus, MMP-9 levels are a useful diagnostic tool for early sepsis detection, which can then inform the subsequent intervention approach.

Recent advances in biosensors, particularly in point-of-care testing (POCT), are pushing towards more individually tailored healthcare delivery [27,28,29]. In 2019, Jundi et al. [30] described a microfluidics-based system [31] that automatically determines clinically significant levels of specific biomarkers for sepsis in approximately 25 min using a finger prick of blood. The researchers found that by assessing leukocyte phenotype and function and measuring leukocyte activation, they could more accurately predict the clinical course of sepsis development in patients. Moreover, this method was more reliable than complete blood-count indicators. Yarbakht et al. [31] described a technique for analyzing early septic liver injury in a murine model of polymicrobial abdominal infection using label-free, nonlinear multimodal imaging combined with coherent anti-Stokes Raman scattering (CARS). They reported that CARS was the most reliable method to distinguish sepsis in a controlled trial, and they concluded that this approach provided reliable diagnostic information relating to morphological and chemical composition changes at the early stages of sepsis. 

Furthermore, Crapnell et al. [32] demonstrated functionalized screen-printed electrodes (SPEs) in conjunction with a thermal detection methodology for rapid diagnosis of sepsis. They presented a sensing system that uses functionalized screen-printed electrodes (SPEs) and a heat-transfer method (HTM) to detect the presence of interleukin-6 (IL-6) in human blood plasma as a biomarker for sepsis detection. The results can be delivered within 15 min following sample injection and provide a graduated diagnostic result, according to the severity of each sepsis case, demonstrating the high value of novel diagnostic development approaches and their clinical value.

Paper is widely utilized as a flexible substrate in developing diagnostic tools because of the ease of fabrication and production of colorimetric assays at low cost and can be used by non-skilled personnel in the field. Moreover, the simplicity of the production of gold and other metallic nanoparticles as well as their intrinsic stability has increased the interest in using AuNPs to interface with biological recognition in a variety of biosensor applications. We have recently developed a paper-based peptide magnetic beads biosensing platform which was successfully used for the detection of some biomarkers for diagnostic applications [33,34]. The detection was based on the cleavage of a specific peptide substrate attached to black magnetic nanoparticles from a gold-coated paper substrate upon binding with the protease analyte. The detachment of the peptide magnetic beads leads to a change in the color of the paper sensor from black to golden yellow color, which can be visualized by the naked eye. This method is, rapid, highly sensitive, low-cost, and easy to perform.

Here, MMP-9 is studied as one of the biomarkers that can be used for the early detection of sepsis. In this study, we present and test a paper-based sensing platform to detect MMP-9 for the first time as a biomarker for sepsis using a fecal intraperitoneal (FIP) approach in a live mouse model. This work provides clear evidence that MMP-9 is a useful biomarker in the detection of sepsis and presents a simple low-cost diagnostic tool that can be exploited to detect other sepsis biomarkers in the future.

## 2. Materials and Methods

### 2.1. Reagents and Materials

The MMP-9 peptide target used for this study was synthesized by Pepmic Co. (Suzhou, Jiangsu, China). Ethyl-3-(3-dimethylaminopropyl)-carbodiimide(EDC), N-hydroxy-succinimide (NHS), and ethylenediaminetetraacetic acid (EDTA) were purchased from Sigma-Aldrich (Darmstadt, Germany). Wash/storage buffer (10 mM Tris base, 0.15 M sodium chloride, 0.1% (*w/v*) bovine serum albumin, 1 mM EDTA, 0.1% sodium azide, pH 7.5) and the coupling buffer (10 mM potassium phosphate and 0.15 M sodium chloride, pH 5.5) were prepared from chemicals of analytical grade. Fetal bovine serum (FBS) and the Diff-Quick stain kit were obtained from Gainland Chemical (Sandycroft, Deeside, UK). TNF-α and IL-6 kits were purchased from R&D system (Minneapolis, MN, USA). Carboxylic acid-modified magnetic nanoparticles (around 50 nm in diameter) were obtained from Turbobeads (Zurich, Switzerland). All reagents and solutions were prepared using deionized water. MMP-9 aliquots were stored at minus 20 °C until further use.

### 2.2. Animal Preparation

Swiss male wild-type (WT) mice (Bar Harbor, ME, USA) aged between 6–12 weeks and weighing 18–25 g were purchased from Jackson Laboratories. The mice were housed in the Department of Animal Facilities at the Saudi Food and Drug Authority (SFDA) under specific pathogen-free conditions with a 12 h light/dark cycle and free access to food and water. Mice were used as a model for sepsis infection and as a subsequent source of blood and Bronchoalveolar Lavage (BAL) fluid samples. All procedures were approved by the Institutional Animal Care and Use Committee at Alfaisal University. Animal care and handling procedures were performed in accordance with the National Institutes of Health guidelines for ethical animal treatment and received IRB approval by King Fahad Medical City NO: FWA00018774.

### 2.3. Induction of Sepsis

We used a non-surgical animal model involving the administration of a parenteral injection of a substance to induce rapid distribution around the body via the venous system and to minimize any additional risks present with surgical interventions. Parenteral injection generally creates a systemic inflammatory response. In this study, we administered an intraperitoneal injection of a pre-prepared fecal solution (FS) to initiate peritonitis in mice, which further developed into sepsis [35,36,37]. We used this two-step challenge (FIP followed by peritonitis) to document how MMP-9 would capture the signs of early and/or severe sepsis in mice after 1 h, 2 h, 4 h, and 20 h periods post-challenge. 

### 2.4. Preparation of Fecal Intraperitoneal (FIP) Model of Sepsis

Fecal material (180 mg) was collected from the lower cecum of six euthanized mice, suspended in 1 mL of 0.9% saline, and filtered to remove particulate matter using a 70 μm nylon mesh strainer (BD Biosciences, Franklin, NJ, USA), thereby yielding FIP administration. We used this freshly prepared FIP and administered it intraperitoneally at pre-prepared concentrations to induce acute peritonitis in experimental mice. FIP (0.5 mL) was injected intraperitoneally with a syringe and 27-gauge needle to induce sepsis (this is an accepted method of successfully inducing sepsis in mice models) [38]. Control mice were given the same volume of sterile normal saline (NS) and were injected in the same location. We used three experimental mice and three control mice. The pre-prepared concentrations of FIP used were 360, 180, 90, 45, 22.5, 11.25, and 5.6 µL/mL.

### 2.5. Intracardiac Puncture for Terminal Blood Collection in Mice

Six hours after FIP injection, plasma (20% dilution) was obtained to probe the inflammatory changes after sepsis induction. Cardiac puncture was applied to collect a large volume of high-quality blood from the experimental mice. Sample collection was performed after FIP administration while mice were in terminal anesthesia. Skin disinfection was ensured by swabbing the skin surface with 70% ethanol twice prior to sample collection from the FIP-treated and control mice, and this process was repeated 1 h, 2 h, 4 h, and 20 h post-challenge. Blood samples were stored at −80 °C until further use.

### 2.6. Bronchoalveolar Lavage Fluid (BALF)

After 24 h of FIP treatment, mice were anesthetized by intraperitoneal injection of a solution containing ketamine (80 mg/kg) and xylazine (5 mg/kg). An incision was made in the mouse’s neck, and the trachea was punctured using a 1 cc needle. Then, the trachea was cannulated with a 24-gauge catheter and secured in place with 40 sutures. The lungs were lavaged with 6–8 aliquots of 0.6 mL sterile PBS. BAL samples were collected and stored at −80 °C until further use.

### 2.7. Cell Counting

Blood samples collected from FIP-treated and control mice were transferred into Eppendorf tubes. A blood smear was made and stained using the Diff-Quick stain kit according to the manufacturer’s directions. Slides were placed in eosin solution for 30 s, methylene blue solution for 30 s, and washed with tap water to remove the residual dye. Neutrophils were identified and counted using a microscope.

### 2.8. Cytokine and MMP-9 Measurements using Enzyme-Linked Immunosorbent Assay (ELISA)

One milliliter of each blood sample was centrifuged at 1200 rpm for 5 min at 25 °C, and serum was separated and stored at −80 °C. To measure the concentrations of TNF-α, IL-6, and MMP-9 in the samples, commercially available kits for the corresponding cytokines were used following the manufacturer’s instructions. Standard ELISA protocol was utilized where the results were detected using TMB and H_2_SO_4_ solution, and the absorbance measurements were recorded at 450 nm as this wavelength was recommended to give the highest sensitivity. Sample absorbance was obtained using a plate reader (R and D ELISA reader, Jena, Germany). Calibration curves of TNF-α and IL-6 were initially constructed in the ranges of 50–500 pg/mL and 500–2000 pg/mL, respectively. All samples were thawed only once and assayed in triplicate.

### 2.9. Immunoblot Analysis

After centrifugation and removal of the insoluble fraction, equal amounts of total protein and EB buffer were used to adjust the total volume. Anti-EE antibodies (2 µg) were added to the lysates with 30 μL of 50% protein A sepharose suspension. The samples were boiled in 2× Laemmli sample buffer for separation with SDS-PAGE. Proteins were transferred into polyvinylidene difluoride (PVDF) membranes (Immobilon-P, Millipore, Watford, UK) for Western Blot analysis. The membranes were probed overnight at 4 °C with the appropriate primary antibody (MMP-9). After washing, the membranes were then probed with horseradish peroxidase-linked secondary antibody for 1 h at room temperature and visualized via enhanced chemiluminescence (ECL).

### 2.10. MMP-9 Magnetic Beads Conjugation

A total of 15 mg of magnetic nanobeads (MNBs) functionalized with carboxylic acid was suspended in water and dispersed by sonication for 5 min and washed with the coupling buffer (10 mM potassium phosphate, 0.15 M sodium chloride, pH 5.5) three times. A total of 1 mg of MMP-9 peptide was dissolved in 1 mL of DMSO. Then, 100 µL of the peptide solution was mixed with 1 mL of the MNBs suspension (15 mg/mL in coupling buffer), EDC (0.57 mg), and NHS (12 µg). The mixture was stirred at room temperature and stored overnight at 4 °C. Uncoupled peptides were removed by gently washing MNBs three times with washing buffer. MNBs-peptides conjugate was stored at 4 °C until further use.

### 2.11. Sensing Platform Preparation

Gold thin film was sputtered in the clean room at King Abdullah University of Science & Technology (KAUST) onto a self-adhesive sheet (Whatman, London, UK). Coated sheets were then cut into 1–2 mm pieces and stacked onto a plastic support at a specific distance. A total of 20 µL of MMP-9 peptide-MNBs suspension was dispensed on the gold-coated sensor surface and kept at room temperature for 30 min. An external NdFeB magnet (10 × 10 × 5 mm) with a magnetic field strength of 3300 gauss at a 2 mm distance was passed over the functionalized strip to remove any unattached peptide-MNBs conjugates. At this stage, the sensor surface color turns black due to the MNB coverage. Then, a round magnetic paper was fixed 2–3 mm beneath the sensing platform. Upon assay operation, the golden color of the sensing platform was restored.

### 2.12. Mice Blood Cultures (BCs)

Whole blood (4 mL) was collected from FIP-treated mice into sterile VACUETTE EDTA K2E tubes (Greiner Bio-One, Frickenhausen, Germany). Each blood sample was cultured in a pair of aerobic/anaerobic Eppendorf tubes. Identification of observed organisms was confirmed by Gram stain and biochemical tests, including oxidase, catalase, coagulase, streptococcus grouping kit, Triple Sugar Iron Agar (TSI), and (Analytical profile index) API test.

### 2.13. Statistical Analysis

Statistical analysis was performed using GraphPad Prism software version 5.04 (San Diego, CA, USA). Data were presented as (mean ± SEM). The student’s *t*-test was used to compare two groups, while ANOVA followed by Bonferroni was used to compare multiple groups. Significance was set at the *p* ˂ 0.05 level.

## 3. Results and Discussion

### 3.1. Evaluation of Different Biomarkers for Sepsis in Infected Mice

**Figure 1:**
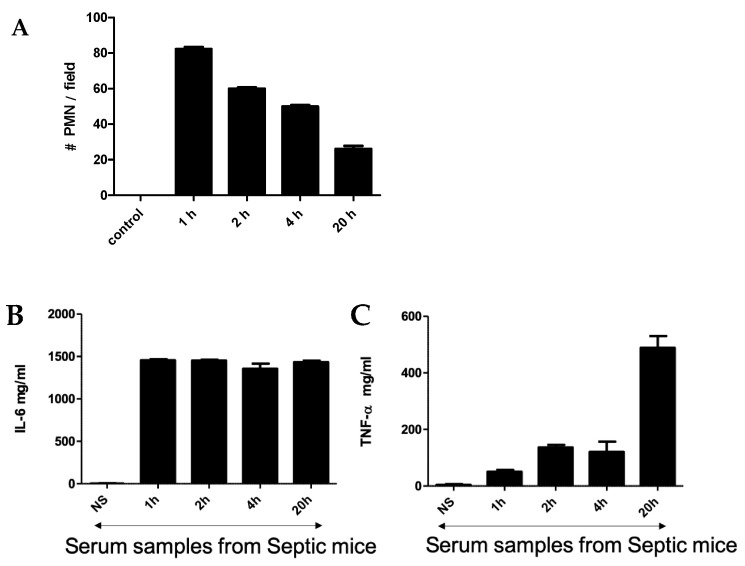
(**A**) Neutrophil cell count in blood smear obtained from mice challenged with FIP. Cytokines (IL-6 (**B**)) and TNF-α (**C**) were measured using ELISA at 1 h, 2 h, 4 h, and 20 h after FIP versus normal saline as control.

An intraperitoneal injection of a pre-prepared fecal solution (FS) was used to initiate peritonitis in mice, which further developed into sepsis. A two-step challenge (FIP followed by peritonitis) was used. First, plasma protein levels of pro-TNF-α and IL-6 as well as polymorphonuclear neutrophils (PMNs) count were determined in FIP-treated mice and compared with control mice. As shown in Figure 1, in the control mice, no PMNs, TNF-α, or IL-6 were detected after injection with normal saline, but they were clearly present in the FIP-treated mice. Figure 1A shows the PMN count from blood samples taken at 1 h, 2 h, 4 h, and 20 h post-challenge. The PMN count was highest at 1 h post-challenge and gradually decreased with time to display the lowest value at 20 h post-challenge. TNF-α and IL-6 in the serum of septic mice were also measured following FIP administration at 1 h, 2 h, 4 h, and 20 h post-challenge. We found that IL-6 was higher than TNF-α through all time intervals (Figure 1B,C). The magnitude of IL-6 expression was steady through the course of the experiment while TNF-α expression was significantly higher at 20 h post-challenge, which was statistically significant (*p* < 0.05). This demonstrates that TNF-α and IL-6 are associated with sepsis in FIP-treated mice and supports previous work that suggests TNF-α and IL-6 upregulation is a hallmark of the early onset of sepsis [39,40,41,42].

### 3.2. Detection of MMP-9 in Infected Mice Using the Magnetic Nanobeads Peptide Paper-Based Platform

**Figure 2:**
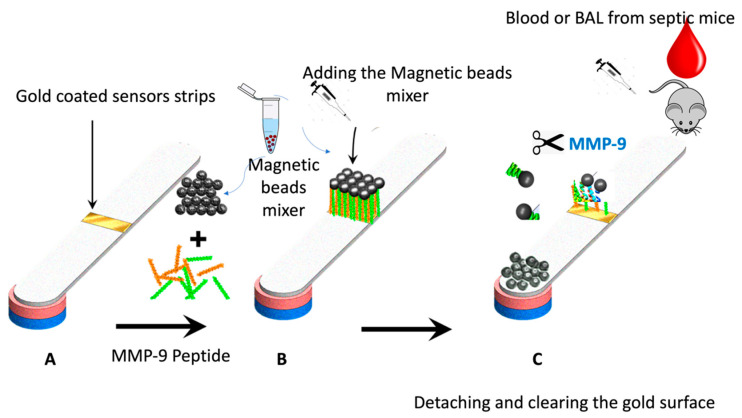
Schematic representation of the working principle of the paper-based sensor for the MMP-9; (**A**) adhesive tape coated with gold was stacked over the paper, (**B**) peptide-conjugated magnetic nanobeads were loaded to the gold surface to form a functional sensor, and (**C**) the FIP-treated blood or BAL samples from the mice was added, exposing the gold-colored surface due to the protease cleavage of the MMP-9 peptide substrate.

Figure 2 shows the paper-based sensor for the MMP-9, which was prepared using the immobilization of magnetic nanobeads-MMP-2 peptide substrate attached to the gold-coated paper substrate. The conjugation of the peptide to the magnetic particle was conducted via covalent bonding of the carboxylic acid groups on the nanobeads surface with the N-terminal of the MMP-9 peptide using the EDC/NHS chemistry. The attachment of the peptide-MNBs conjugate to the gold-coated paper substrate, led to the formation of a black layer on the gold surface, which can be clearly visualized by the naked eye. The addition of the blood and BAL samples containing MMP-9 collected from the infected mice caused a cleavage of the peptide sequence led to the detachment of the MNBs from the paper sensor surface. The detached particles are attracted using the magnetic paper placed underneath the paper substrate.

**Figure 3:**
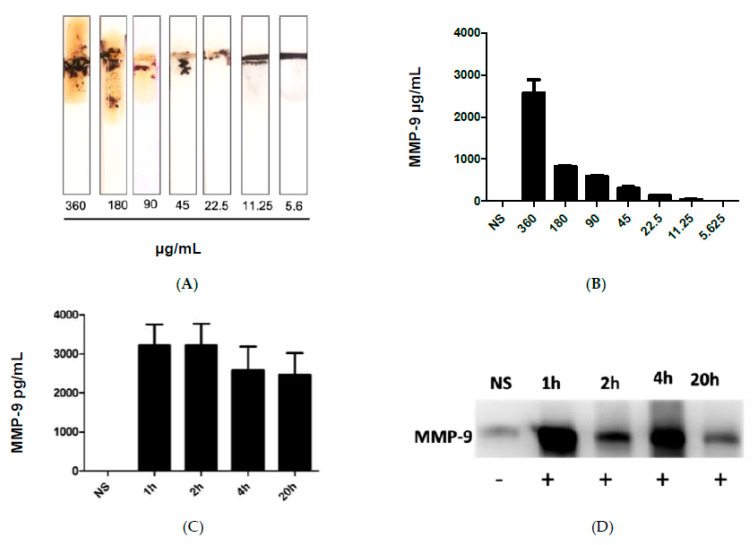
(**A**) image of the MMP-9 protease sensor responses for different blood samples taken from injected mice with different fecal concentrations. (**B**) Quantitative response of the MMP-9 concentration, *n* = 3. (**C**) MMP-9 levels detected using ELISA in mice and blood obtained from mice treated with FIP at 1 h, 2 h, 4 h, and 20 h post-challenge. (**D**) Western blot for the measurement of MMP-9 in blood samples of infected mice collected at different time intervals.

Figure 3A shows the results after the addition of the blood samples collected from infected mice with different administered fecal concentrations, which in turn caused cleavage of the peptide-MNBs conjugate, leading to the appearance of the gold surface and the change of the color. MMP-9 levels were highest at the most concentrated levels of administered FS, whereas no change was observed for the control sample. Figure 3B shows the quantitative measurements conducted using ImageJ software1.51 version 2015. Figure 3C shows the quantification of MMP-9 at 1 h, 2 h, 4 h, and 20 h post-challenge using ELISA. Western Blot was performed for the same samples for comparison (Figure 3D). The Western Blot results shown in Figure 3D were in agreement with the sensor results.

To study the specificity of our paper-based sensor, we have studied the cross reactivity against other biomarkers related to inflammatory diseases including sepsis such as IL-6 and TNF-α. However, no significant cross-reactivity was detected indicating the specificity of the sensor to MMP-9.

**Figure 4:**
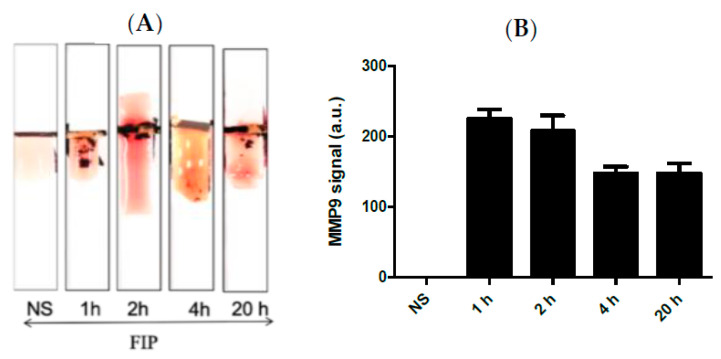
(**A**) Representative images and (**B**) quantitative response of the paper-based biosensors for rapid detection of MMP-9 in blood samples obtained from septic mice at different time intervals treated with FIP and controls treated with NS, *n* = 3. The chart bar graphs represent the quantitative data.

Figure 4A shows the results of the MPP-9 paper-based sensors for blood samples obtained from septic mice at different time intervals treated with FIP and controls treated with NS. It was found that the MMP-9 concentration increased immediately post-challenge and decreased thereafter up to the 20 h post-challenge window. The results of the paper-based sensor were in good agreement with the results obtained with ELISA and Western Blot. This indicates that the MMP-9 peptide biosensor has potential as an early-stage sepsis sensor. High concentrations of MMP-9 post-challenge support the conclusions of the previous studies, which documented that the MMP-9 levels rise in relation to an immunological response to infection [12,13,14,17,18,19].

**Figure 5:**
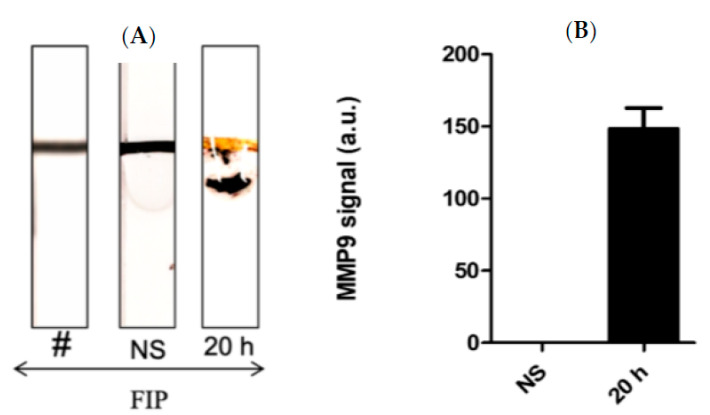
(**A**) Representative images and (**B**) the quantitative response of the paper-based biosensors for rapid detection of MMP-9 in BAL samples obtained from septic mice at different time intervals treated with FIP and controls treated with NS, *n* = 3. The chart bar graphs represent the quantitative data.

Kumar et al. [43] demonstrated that BAL samples can provide a reliable source of cellular and acellular components with minimally invasive collection procedures. In this work, a series of Bal samples collected at different time intervals were tested using the MMP-9 paper–based sensors. It was found that BAL samples taken at 20 h post-challenge confirmed a higher concentration of MMP-9 in comparison with the control mice samples (Figure 5A,B). We found that the FIP model successfully induced sepsis in all experimental mice and mimics the pro-inflammatory response observed in clinical sepsis. Triggering sepsis in the experimental mice caused an increase in the MMP-9 concentration in blood and BAL samples (Figure 4 and Figure 5). MMP-9 levels were highest after the challenge with the most concentrated FS and were higher within the time periods closest to the challenge administration window, indicating that MMP-9 production is rapid and highly correlated with the onset of sepsis. Additionally, we found bacterial growth in every major organ in the septic mice.

It is worth mentioning that the bacterial testing of the blood cultures at 1 h and 2 h post-challenge reflected the presence and growth of Gram-positive bacteria, including *Streptococcus’s* group D and *Staphylococcus. SPP.* However, testing the blood samples at 4 h and 20 h post-challenge indicated the growth of Gram-negative bacteria, including *E. coli* SPP and *Salmonella* serotype O, confirming the successful induction of sepsis. It is worth mentioning that not all anaerobic microorganisms present in the samples were identified. We observed colonies that varied in size, shape, and color, which suggests a highly polymicrobial nature of the infection. 

## 4. Conclusions

In this study, we detected the MMP-9 protease released from a FIP mice model. MMP-9 paper-based sensors fabricated and demonstrated their capability to detect the MMP-9 levels in both blood and BAL samples in sepsis-challenged mice as early as 1 h and up to 20 h post-challenge. The paper-based sensor was based on the cleavage of the peptide-magnetic nanoparticles conjugated to MMP-9 peptide substrate and attached to the sensor surface. Upon exposure to the MMP-9 protease, it will reveal the gold color of the sensor surface initially covered with black beads. The results were in good agreement when compared and validated with Western Blot and ELISA. Thus, we conclude that MMP-9 can be used as an effective biomarker for sepsis and could detect the disease at all stages, thus providing very useful information about the sepsis patient’s status. The reported paper-based assay can provide rapid diagnosis showing potential advantages over existing traditional methods in terms of simplicity cost reductions, and fast response. In addition, the detection method can be read by the naked eye in the field without the need for laboratory-based equipment. 

Future research seeks to replicate our findings in human studies to confirm its clinical viability and application in the healthcare system.

## Data Availability

Not applicable.
